# An Autonomous Pathogen Detection System for Assessing Temporal Associations with Respiratory Pathogen Activity

**DOI:** 10.3390/microorganisms14102192

**Published:** 2026-10-01

**Authors:** Yingying Liu, Na Wang, Yifei Wang, Kexin Zhao, Huipeng Li, Yongxue Yan, Fanshuang Li, Renjie Yang, Sitong Liu, Ziqi Xie, Qijia He, Yu Niu, Kongxin Hu

**Affiliations:** Institute of Synthetic Biology Product Quality and Safety (Secretariat of the Biosafety Coordination Mechanism of SAMR), Chinese Academy of Quality and Inspection & Testing, Beijing 100176, China

**Keywords:** respiratory pathogen aerosols, airborne transmission, autonomous triggering, in situ detection, time-series correlation analysis

## Abstract

Current respiratory pathogen surveillance is largely based on case reporting, sentinel surveillance, and environmental sampling. This study developed an autonomous pathogen detection system (APDS) integrating real-time bioaerosol sensing, intelligent triggering, aerosol collection, nucleic acid extraction, real-time PCR, and automated data reporting for respiratory pathogen surveillance. System performance was evaluated in a 20 m^3^ aerosol chamber and through 52 weeks of continuous monitoring in a hospital outpatient hall in Beijing. Laboratory experiments showed that the developed platform enables the detection of airborne influenza A virus at concentrations as low as 3.75 × 10^4^ 50% tissue culture infectious dose/m^3^ and can detect airborne *Pseudomonas aeruginosa* at concentrations as low as 1.80 × 10^4^ copies/m^3^. The platform can detect low concentrations of airborne pathogen nucleic acids that are undetectable by surface sampling. During field monitoring, APDS autonomously triggered 381 tests and recorded 46 pathogen-positive detections. Respiratory syncytial virus showed significant correlations when APDS signals preceded national surveillance data by 1–3 weeks, with the strongest correlation at a 3-week lead (ρ = 0.487). Given the spatial mismatch between single-site APDS monitoring and national surveillance data, this association should be considered exploratory. These findings demonstrate the feasibility of APDS for long-term automated aerosol surveillance and suggest its potential as a complementary tool for respiratory pathogen monitoring.

## 1. Introduction

Respiratory pathogens can enter the air in respiratory particles released by infected individuals during breathing, coughing, or sneezing. Small pathogen-containing aerosols can remain suspended and disperse in the air for extended periods, enabling transmission over both short and longer distances [1]. Previous studies have shown that SARS-CoV-2, influenza viruses, rhinoviruses, and other pathogens can be transmitted through bioaerosols, with transmission risk increasing particularly in poorly ventilated, crowded indoor environments [2].

Timely identification of airborne pathogens is important for the early recognition of transmission risk and the implementation of interventions. Current surveillance of respiratory infectious diseases relies mainly on case reporting, clinical sentinel surveillance, and environmental surface sampling [3], and the resulting information may lag behind infection or environmental contamination. Air sampling combined with molecular methods such as qPCR has improved the detection of bioaerosols; however, sampling, transport, pretreatment, and testing are typically performed as separate steps, resulting in relatively long workflows. In addition, conventional scheduled sampling cannot respond in real time to changes in environmental risk and may therefore lead to unnecessary sampling or missed transmission signals.

Current airborne microbial sampling methods mainly collect aerosols by gravitational settling, cascade impaction, electrostatic precipitation, or centrifugation, followed by identification using culture-based or molecular methods [4,5,6,7,8]. Conventional culture methods are time-consuming, whereas chromatographic and mass spectrometric methods are limited by instrument cost, operational complexity, and requirements for field deployment. Therefore, respiratory pathogen surveillance would benefit from automated technologies capable of sensing changes in biological particles in real time, autonomously triggering sampling, and completing in situ detection to improve timeliness and early risk recognition [9,10,11,12].

In this study, we developed an in situ autonomous pathogen detection system (APDS) that integrates optical sensing with molecular diagnostics to enable biological-particle monitoring, intelligent triggering of aerosol sampling, and in situ detection of pathogen nucleic acids. First, the detection sensitivity of APDS was evaluated in an aerosol chamber and compared with conventional surface sampling. We then conducted 52 weeks of continuous monitoring in a hospital outpatient hall and compared the results with data from national sentinel surveillance of acute respiratory infectious diseases to assess monitoring performance for different respiratory pathogens, examine temporal associations with national surveillance data, and explore the potential for early indication. This study aimed to investigate an automated approach for monitoring airborne transmission risk from respiratory pathogens and to provide a technical basis for the integrated use of environmental and clinical surveillance data.

## 2. Materials and Methods

### 2.1. System Design and Operating Principle

APDS comprises three modules—real-time sensing, intelligent decision-making, and in situ detection—forming a closed-loop workflow from environmental monitoring and risk identification to pathogen detection.

The real-time sensing module includes biological and chemical sensing units. The biological sensing unit uses laser-induced fluorescence (LIF) to continuously monitor total particles and fluorescent particles in the 0.5, 1.0, and 5.0 μm size channels, as well as biological particles in the 1.0 and 5.0 μm size channels. The chemical sensing unit simultaneously monitors environmental parameters including temperature, relative humidity, CO_2_, formaldehyde, and volatile organic compounds (VOCs).

The intelligent decision-making module analyzes sensor data in real time. When the biological-particle count and CO_2_ concentration remain above predefined thresholds within a specified time window, the system identifies the condition as a risk event and automatically triggers testing, with a response time of less than 30 s. The thresholds can be adjusted according to the application scenario.

After triggering, the in situ detection module activates a gas–liquid mixing air-sampling device [9] to transfer aerosol particles into the liquid phase and enrich target pathogens using in-house-developed broad-spectrum conjugated magnetic beads, achieving greater than 10-fold enrichment [13]. The air-collection principle is consistent with that of the BIO Capturer-6 viral aerosol collection and enrichment instrument [14]. The system then automatically performs nucleic acid extraction, purification, and multi-target real-time PCR detection. The time from triggering to result output is 20 min–2 h. After detection, the system automatically replaces reagents and consumables and uploads the results to the monitoring cloud platform. All nucleic acid testing kits were purchased from Beijing Applied Biological Technologies Co., Ltd. (Beijing, China). The specific kits and catalog numbers were as follows: Influenza A virus, A3011YH-50T; *Streptococcus pneumoniae*, A5531YH-50T; *Pseudomonas aeruginosa*, A3541YH-50T; SARS-CoV-2, CT8233-48T; *Legionella pneumophila*, 5971YH-50T; human rhinovirus, A3891YH-50T; respiratory syncytial virus, A3931YH-50T; and enterovirus, A2691YH-50T.All procedures were performed strictly in accordance with the manufacturer’s instructions.

### 2.2. Experimental Design and Performance Validation

#### 2.2.1. Reagents and Instruments

The experiments used an influenza A virus quality-control material (370051–201801, National Institutes for Food and Drug Control, Beijing, China) with a titer of 1.00 × 10^7^ TCID_50_/mL; a SARS-CoV-2 quality-control material with an assigned in-house titer of 5.13 × 10^6^ TCID_50_/mL. Both viral quality-control materials were heat-inactivated at 56 °C for 30 min before use; a *Streptococcus pneumoniae* quality-control material (BNCC363536, Henan Provincial Engineering Research Center for Industrial Microbial Culture Collection, Xinyang, China) at 5.30 × 10^7^ copies/mL; a *Pseudomonas aeruginosa* quality-control material (BNCC365272, Henan Provincial Engineering Research Center for Industrial Microbial Culture Collection) at 1.20 × 10^7^ copies/mL; and a *Legionella pneumophila* quality-control material (BNCC373318, Henan Provincial Engineering Research Center for Industrial Microbial Culture Collection) at 5.10 × 10^7^ copies/mL. All quality-control materials described above were non-infectious.

#### 2.2.2. Aerosol Chamber Experiments

A 20 m^3^ aerosol chamber (3.1 m × 2.5 m × 2.6 m) equipped with ventilation, gas-line, and automated control systems was used to regulate temperature and humidity, generate and mix aerosols, and disinfect the chamber after each experiment. Before each experiment, APDS was placed in the chamber for real-time monitoring of aerosol changes and online in situ detection and identification of pathogenic microorganisms. For each experiment, 9 mL of the test suspension was added to the microbial aerosol generator, of which 3 mL was nebulized. The generator was positioned 1.5 m above the floor, filtered air was used as the nebulizing gas, and airflow was controlled using a mass flow controller. Nebulization was performed for 5 min, followed by 10 min of mixing with a dispersal fan. During the experiments, the chamber temperature was maintained at 25 °C and the relative humidity was set to either 20% or 70%.

#### 2.2.3. Determination of the Minimum Consistently Detectable Concentration of Respiratory Pathogen Aerosols

The test suspensions were serially diluted 10-fold in PBS, and 9 mL of each dilution was added to the aerosol generator for nebulization. After aerosol mixing was completed, the APDS detection workflow was manually initiated, after which the system automatically performed air sampling, target-pathogen enrichment, nucleic acid extraction, and real-time PCR detection. Because the aerosol chamber experiments were designed primarily to evaluate aerosol collection and in situ detection performance, the autonomous triggering strategy based on biological-particle and CO_2_ concentrations was disabled.

The theoretical aerosol concentration of each pathogen in the chamber was calculated as C = (c × v)/V, where C is the theoretical aerosol concentration in the chamber, c is the concentration of the target pathogen in the nebulized suspension, v is the actual nebulized volume, and V is the chamber volume (20 m^3^). This concentration was calculated from the amount of target pathogen introduced and the chamber volume and was not corrected for aerosol-generation efficiency, tubing losses, particle settling, or wall deposition. It was therefore used to represent the theoretical exposure level under different experimental conditions and does not represent the actual aerosol concentration quantified by independent air sampling. The lowest theoretical aerosol concentration that yielded positive results in all three replicate tests was defined as the minimum consistently detectable concentration under the conditions of this study. The experimental conditions are summarized in Table 1. The layout of environmental monitoring locations is provided in the Supplementary Materials.

### 2.3. Field Monitoring Study

APDS was deployed in the outpatient hall of a medical institution in Beijing and operated continuously to collect monitoring data from weeks 1–52 of 2025. During the study, the system operated in routine mode without manual intervention other than replacement of reagents and consumables. APDS results were compared with national sentinel surveillance data for acute respiratory infectious diseases released by the Chinese Center for Disease Control and Prevention to assess concordance in pathogen detection and temporal trends and to analyze the temporal relationship between environmental and clinical surveillance signals. A multi-target real-time PCR panel was used to detect five respiratory pathogens: influenza virus, respiratory syncytial virus, SARS-CoV-2, rhinovirus, and enterovirus. Each target was detected in a separate reaction well using pathogen-specific primers and probes. Multiple reaction wells were run in parallel during each triggered test, allowing different pathogens to be detected within the same triggered test if present. Each triggered test served as the basic recording unit, and data were aggregated by week. Positive or negative results were recorded separately for each target pathogen in every triggered test, and multiple pathogens could be positive in the same test. The weekly positivity rate for each pathogen was defined as the number of positive detections for that pathogen divided by the total number of APDS-triggered tests performed during the same week. The weekly numbers of triggered tests and pathogen-specific positive detections are provided in Supplementary Materials.

### 2.4. Statistical Analysis

All statistical analyses were performed using R software (version 4.5.2; R Foundation for Statistical Computing, Vienna, Austria). Experimental data are presented as the mean ± standard deviation (mean ± SD). Detection results obtained at 20% and 70% relative humidity were compared using paired *t*-tests. Spearman rank correlation analysis was used to evaluate temporal associations between APDS and national sentinel surveillance data over time lags ranging from −3 to +3 weeks. Lag = 0 indicates a concurrent comparison; a positive lag indicates that the APDS environmental surveillance signal precedes the national sentinel surveillance signal, whereas a negative lag indicates that it follows the national signal. For each pathogen, the seven lag-specific correlation tests were adjusted using the Benjamini–Hochberg method to control the false discovery rate (FDR), and an adjusted q < 0.05 was considered statistically significant.

Monitoring weeks were classified as APDS-positive or APDS-negative according to whether the target pathogen was detected. The corresponding national sentinel surveillance positivity rates were compared between the two groups using the Mann–Whitney U test. All statistical tests were two-sided. Except for the time-lag correlation analysis, for which FDR-adjusted q values were used, *p* < 0.05 was considered statistically significant.

## 3. Results

### 3.1. Real-Time Online Monitoring of Airborne Particles in the Laboratory

Aerosol chamber experiments were conducted in a 20 m^3^ chamber at 25 °C and relative humidities of 20% and 70%. Under both humidity conditions, the concentrations of 0.5 μm and 1.0 μm particles increased rapidly after aerosol generation began and peaked at approximately 7–8 min, whereas the concentration of 5.0 μm particles remained relatively low. After aerosol generation was stopped, particle concentrations in all size ranges gradually decreased over time. At 20% relative humidity, the 0.5 μm and 1.0 μm particles had largely settled within approximately 5 h; at 70% relative humidity, some suspended particles remained detectable in the chamber after 6 h. Overall, particle concentrations showed similar temporal patterns under the two humidity conditions, although smaller particles remained suspended for longer at 70% relative humidity (Figure 1).

### 3.2. Minimum Consistently Detectable Concentrations of Respiratory Pathogen Aerosols in the Laboratory

To evaluate the ability of APDS to detect respiratory pathogen aerosols, mixed quality-control materials containing influenza A virus, Streptococcus pneumoniae, and Pseudomonas aeruginosa were aerosolized at different concentrations at 25 °C and relative humidities of 20% and 70%. Under the M1 condition, all three target pathogens were detected at both relative humidity levels. Under the M2 condition, influenza A virus and Pseudomonas aeruginosa remained detectable at both relative humidity levels, whereas none of the target pathogens were detected under the M3 condition. Based on three consecutive replicate tests, the minimum theoretical aerosol concentration that can be consistently detected under operating conditions by APDS was 3.75 × 10^4^ TCID_50_/m^3^ for influenza A virus, 7.95 × 10^5^ copies/m^3^ for Streptococcus pneumoniae, and 1.80 × 10^4^ copies/m^3^ for Pseudomonas aeruginosa (Table 2). Further details are provided in the Supplementary Materials.

To further assess the applicability of APDS to different types of respiratory pathogens, SARS-CoV-2 and *Legionella pneumophila* quality-control materials were used to evaluate system performance. When the aerosol concentrations reached at least 7.70 × 10^3^ TCID_50_/m^3^ and 7.65 × 10^4^ copies/m^3^, respectively, APDS consistently detected SARS-CoV-2 and *Legionella pneumophila*, further indicating that the system can perform in situ detection of aerosols containing a range of respiratory pathogens (Table 3).

### 3.3. Comparison of APDS Air Monitoring and Environmental Surface Sampling in the Laboratory

To compare in situ air detection with environmental surface sampling, swab samples were collected from five floor locations and two wall locations after the aerosol chamber experiments (Figure 2).

Under the M1 condition, influenza A virus was detected mainly in floor surface samples: G1–G4 were positive at 20% relative humidity, whereas only G1 was positive at 70% relative humidity. No surface samples were positive for influenza A virus under the M2 or M3 conditions. *Streptococcus pneumoniae* was negative at all surface locations under all experimental conditions. *Pseudomonas aeruginosa* was detected at multiple floor locations under the M1 condition: G1–G4 were positive at 20% relative humidity and G1–G5 were positive at 70% relative humidity. No surface locations were positive under the M2 condition. Target pathogens were not detected at any wall location under any experimental condition.

### 3.4. Overview of Field Monitoring

From weeks 1–52 of 2025, APDS operated continuously in the outpatient hall of a hospital in Beijing. During the year, APDS autonomously triggered 381 tests and yielded 46 pathogen-positive detections, corresponding to an overall positive detection rate of 12.07%. All 46 positive triggered tests contained a single pathogen-positive result, and no test showed simultaneous detection of more than one pathogen. The individual positivity rates for the five pathogens ranged from 1.84% to 3.15%, with respiratory syncytial virus having the highest detection rate (3.15%) and SARS-CoV-2 the lowest (1.84%). Overall, all pathogens were detected at low frequency and intermittently by APDS (Figure 3).

### 3.5. Temporal Trends and Time-Lag Association Analysis

APDS data were compared weekly with national sentinel surveillance data for acute respiratory infectious diseases. Positivity rates for the five pathogens in the national surveillance data varied continuously over time, whereas the threshold-triggered APDS measurements produced intermittent positive signals. APDS-positive signals for influenza virus and respiratory syncytial virus were concentrated mainly in autumn and winter, broadly corresponding to periods of higher activity in the national surveillance data. Environmental detections of enterovirus were concentrated mainly in summer and showed a seasonal pattern similar to that of the national surveillance data. In contrast, SARS-CoV-2 and rhinovirus detections by APDS were more sporadic and showed weaker temporal concordance with national surveillance trends (Figure 4).

A bidirectional time-lag analysis from −3 to +3 weeks was further performed to evaluate the temporal relationship between the two surveillance signals, and the multiple tests across lag conditions were FDR-adjusted for each pathogen (Table 3). Respiratory syncytial virus showed the clearest positive-lag association. No statistically significant correlation was observed at negative lags or at Lag = 0, whereas significant positive correlations were observed when the APDS signal preceded national sentinel surveillance by 1–3 weeks. The correlation coefficient increased with the lag and was highest at Lag = +3 (ρ = 0.487).

Influenza virus showed positive correlations across a relatively broad range of lags. After FDR adjustment, the correlations remained statistically significant at Lag = −2, −1, 0, +2, and +3. Because significant correlations occurred at negative and positive lags as well as at Lag = 0, influenza virus did not show a clear unidirectional leading relationship; rather, the findings mainly reflected temporal concordance between APDS and national sentinel surveillance data over a broader time window.

No statistically significant correlation was observed for SARS-CoV-2 at any lag from −3 to +3 weeks. For rhinovirus and enterovirus, some unadjusted analyses yielded *p* < 0.05, but none remained statistically significant after FDR adjustment, indicating no stable time-lag association pattern (Table 4).

The 52 monitoring weeks were classified as APDS-positive or APDS-negative according to whether the target pathogen was detected. Mann–Whitney U tests showed that the corresponding national sentinel surveillance positivity rates for influenza virus, respiratory syncytial virus, and enterovirus were higher during APDS-positive weeks than during APDS-negative weeks (*p* < 0.05), indicating a statistical association between environmental detection status and concurrent population-level pathogen activity for these three pathogens. No statistically significant differences were observed for SARS-CoV-2 or rhinovirus (*p* > 0.05) (Figure 5).

## 4. Discussion

Infected individuals can continuously release respiratory particles of different sizes during breathing, speaking, and coughing, and some of these particles can carry pathogens [15]. Conventional bioaerosol monitoring generally requires air sampling followed by transfer of the samples to a laboratory for nucleic acid extraction and molecular testing, with sampling and detection performed as separate steps [16,17]. Previous studies have used different types of air samplers combined with PCR or other detection methods to detect airborne pathogens such as *Legionella pneumophila* and influenza virus, although most approaches still rely on subsequent manual processing [18,19]. The APDS developed in this study integrates environmental sensing, autonomous triggering, aerosol collection and enrichment, nucleic acid extraction, real-time PCR, and result uploading into a single workflow, thereby reducing sample transport and manual handling. The aerosol chamber experiments further showed that the system can perform in situ detection of aerosols containing multiple viral and bacterial pathogens, providing an experimental basis for long-term automated monitoring in real-world environments.

Larger particles generally settle more rapidly, whereas smaller particles can remain airborne for longer periods and disperse with airflow. In the aerosol chamber experiments, 0.5 μm and 1.0 μm particles remained detectable for several hours after aerosol generation stopped, whereas the concentration of 5.0 μm particles was relatively low, consistent with established understanding of the suspension and settling behavior of particles of different sizes [20,21,22]. Overall particle-concentration trends were similar under the two humidity conditions, but smaller particles persisted for longer at 70% relative humidity, suggesting that humidity may influence aerosol particle dynamics. These results indicate that APDS can continuously record dynamic changes in aerosol particle concentrations under controlled conditions and provide environmental information for subsequent triggered sampling and pathogen detection.

The comparison between air and surface monitoring further illustrates that the two environmental sampling approaches capture different types of information. At higher aerosol concentrations, positive surface results for influenza A virus and *Pseudomonas aeruginosa* were found mainly at floor locations near the aerosol-generation area, whereas wall locations were generally negative. As aerosol concentrations decreased, some target pathogens remained detectable in air by APDS even when concurrent surface samples were negative. *Streptococcus pneumoniae* similarly showed air detection in the absence of surface detection. These differences may be explained by three factors. First, at low airborne pathogen concentrations, the number of particles deposited on surfaces may fall below the detection limit of swab sampling, while residual airborne signals remain detectable [23]. Second, after deposition, pathogens may be more rapidly affected by desiccation, oxidation, and surface properties, leading to nucleic acid degradation or inactivation [24]. Third, surface swabs may collect microorganisms, organic matter, and other contaminants that interfere with nucleic acid extraction and PCR amplification [25]. In contrast, liquid-phase collection and enrichment in APDS may reduce background interference and improve the detection of low-abundance pathogens. Thus, air and surface monitoring do not provide equivalent information. Air monitoring can complement surface monitoring by identifying nucleic acid signals from target pathogens that remain airborne at the time of sampling.

Field monitoring was further used to evaluate the long-term performance of APDS in a real-world environment. The laboratory aerosol chamber experiments were designed mainly to validate the overall APDS detection workflow and its ability to detect aerosols containing different types of pathogens; therefore, representative viral and bacterial quality-control materials were selected for performance evaluation. For field monitoring, a predefined multiplex real-time PCR panel was used to continuously test for influenza virus, respiratory syncytial virus, SARS-CoV-2, rhinovirus, and enterovirus according to respiratory pathogen surveillance needs. Compared with previous studies of airborne pathogen surveillance that relied mainly on manually scheduled sampling [23,26], APDS was operated continuously for 52 weeks, and the temporal relationship between environmental surveillance signals and national sentinel surveillance data for acute respiratory infectious diseases was further analyzed.

It should be noted that the two surveillance systems reflect pathogen activity at different levels. APDS records nucleic acid detection of target pathogens in ambient air under triggered sampling conditions, with the number of triggered tests during the same period as the denominator. In contrast, national sentinel surveillance reflects pathogen positivity in clinical respiratory specimens from symptomatic patients seeking medical care, with the number of respiratory specimens from influenza-like illness cases tested during the same period as the denominator. The two systems differ substantially in the monitored population, sampling source, triggering mechanism, denominator definition, and data-acquisition process (Table 5); therefore, their absolute positivity rates should not be interpreted as directly equivalent. Temporal trends and their associations are more appropriate for evaluating the relationship between environmental surveillance signals and population-level pathogen activity. This positioning is consistent with the WHO concept that respiratory infectious disease surveillance should combine multiple fit-for-purpose surveillance systems that provide complementary information [27].

Among the five pathogens, respiratory syncytial virus showed the clearest positive-lag association. When the APDS environmental signal preceded national sentinel surveillance by 1–3 weeks, the correlations were positive and remained statistically significant after FDR adjustment, with the strongest correlation at Lag = +3. This finding suggests that, within the setting and monitoring period of this study, environmental detection of respiratory syncytial virus nucleic acid preceded the national sentinel surveillance signal to some extent and may have potential value for earlier indication. However, the APDS data were obtained from a single medical institution in Beijing, whereas the reference data were derived from national sentinel surveillance, resulting in a mismatch in spatial scale and monitored populations. It is therefore not possible to determine whether the observed time difference primarily reflects earlier capture of pathogen activity by environmental monitoring or differences between regional and national epidemic timing. The finding should consequently be regarded as an exploratory temporal association and does not establish a definitive predictive capability of APDS. A previous study likewise reported an association between respiratory virus detection in hospital air and clinical surveillance data [26]. Respiratory syncytial virus can be released into the air in virus-containing respiratory particles [28,29], providing a biological basis for complementary aerosol surveillance; however, the positive time lead observed in this study requires further validation using contemporaneous clinical surveillance data from the same region.

Influenza virus showed a different temporal association pattern from respiratory syncytial virus. Statistically significant correlations were distributed across multiple negative, concurrent, and positive lags and remained after FDR adjustment. Therefore, rather than indicating a clear unidirectional leading relationship, these findings more strongly support temporal concordance between the APDS environmental signal and national sentinel surveillance data over a relatively broad time window. In contrast, SARS-CoV-2 showed no stable temporal association across lags from −3 to +3 weeks. Although the unadjusted correlations for rhinovirus and enterovirus reached statistical significance at some lags, none remained significant after adjustment for multiple comparisons; therefore, no evidence of a stable leading or lagging pattern was obtained. Differences in temporal association patterns among pathogens may be influenced by multiple factors, including pathogen-specific environmental release characteristics, seasonal circulation patterns, environmental stability, monitoring location, and the frequency of environmental detection.

Previous studies have also suggested that air monitoring in densely occupied settings such as hospitals and campuses has the potential to reflect trends in respiratory pathogen activity [30,31]. In addition, the Mann–Whitney U test showed that the corresponding national sentinel positivity rates for influenza virus, respiratory syncytial virus, and enterovirus were higher during APDS-positive weeks than during APDS-negative weeks, further supporting an association between environmental detection status and concurrent population-level pathogen activity for these pathogens. No similar differences were observed for SARS-CoV-2 or rhinovirus. The relatively limited number of environmental positive detections for the latter two pathogens may have reduced the statistical stability of the group comparisons, and longer-term and larger-scale monitoring is needed for further validation.

This study has several limitations. First, the APDS environmental surveillance data were obtained from a single medical institution in Beijing, whereas the clinical reference data were derived from national sentinel surveillance of acute respiratory infectious diseases. Because the two data sources were not strictly matched in spatial scale or monitored population, the observed temporal associations can only reflect overall temporal trends and should not be interpreted as direct temporal correspondence between environmental pathogen activity and patient infections within the same region. In addition, the number of APDS-positive weeks was limited for some pathogens, which may have affected the statistical stability of the time-lag correlation analyses and group comparisons. PCR positivity indicates the presence of pathogen nucleic acids but does not confirm pathogen viability or infectivity. Future studies should incorporate contemporaneous clinical case data from the deployment site or the same region, expand the number of monitoring locations and the monitoring duration, and further optimize the autonomous triggering thresholds to validate the temporal relationship between APDS environmental signals and clinical pathogen activity and to assess the stability and reproducibility of its potential for early indication.

## 5. Conclusions

This study developed an autonomous pathogen detection system (APDS) that integrates environmental sensing, intelligent triggering, aerosol collection and enrichment, nucleic acid detection, and automated result uploading, and evaluated its performance through aerosol chamber experiments and 52 weeks of field monitoring. Laboratory results showed that APDS can perform in situ detection of aerosols containing multiple respiratory viruses and bacteria and, under some low-concentration conditions, can detect target pathogens that are not identified by surface sampling, demonstrating the complementary value of air monitoring for suspended pathogen-containing aerosols. During field monitoring, APDS supported sustained long-term operation and captured temporal patterns of different respiratory pathogens. The observed RSV lag association should be considered exploratory because of the spatial mismatch between single-site APDS monitoring and national surveillance data. These findings provide a feasible technical approach for automated and continuous surveillance of respiratory pathogen aerosols in the environment. APDS may complement existing clinical sentinel surveillance and provide a technical basis for an integrated respiratory infectious disease surveillance framework combining environmental and clinical data.

## Figures and Tables

**Figure 1:**
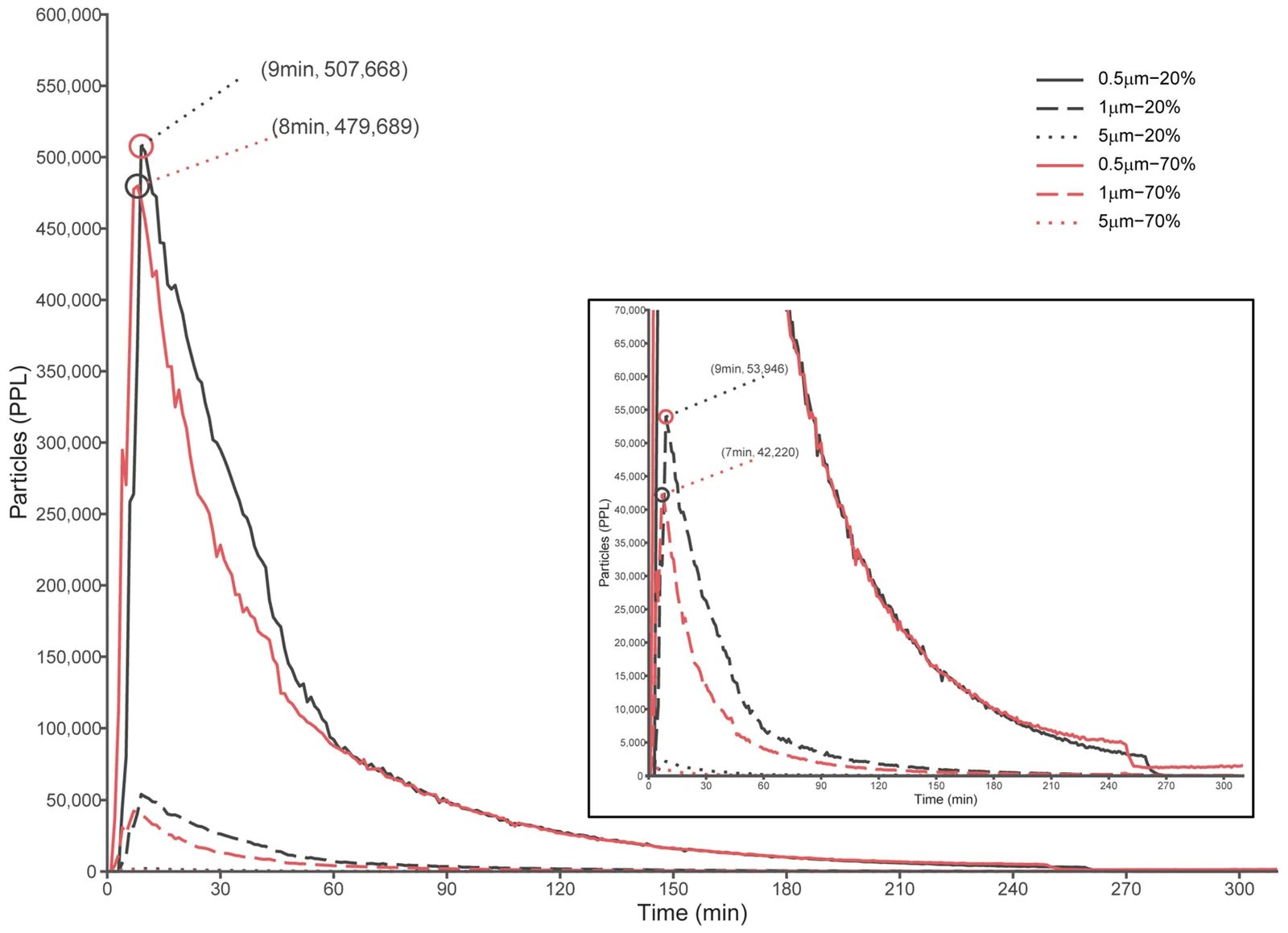
Temporal profiles of aerosol particle concentrations under different relative humidity conditions in the aerosol chamber. Particle concentrations in the 0.5, 1.0, and 5.0 μm size channels were continuously monitored in a 20 m^3^ aerosol chamber at 25 °C under 20% and 70% relative humidity (RH). Black and red curves represent 20% and 70% RH, respectively; solid, dashed, and dotted lines represent particles in the 0.5, 1.0, and 5.0 μm size channels, respectively. Circled annotations indicate the observed peak time (min) and particle concentration (particles per liter, PPL). The inset enlarges the lower-concentration range to facilitate visualization of the temporal profiles of the 1.0 and 5.0 μm particles.

**Figure 2:**
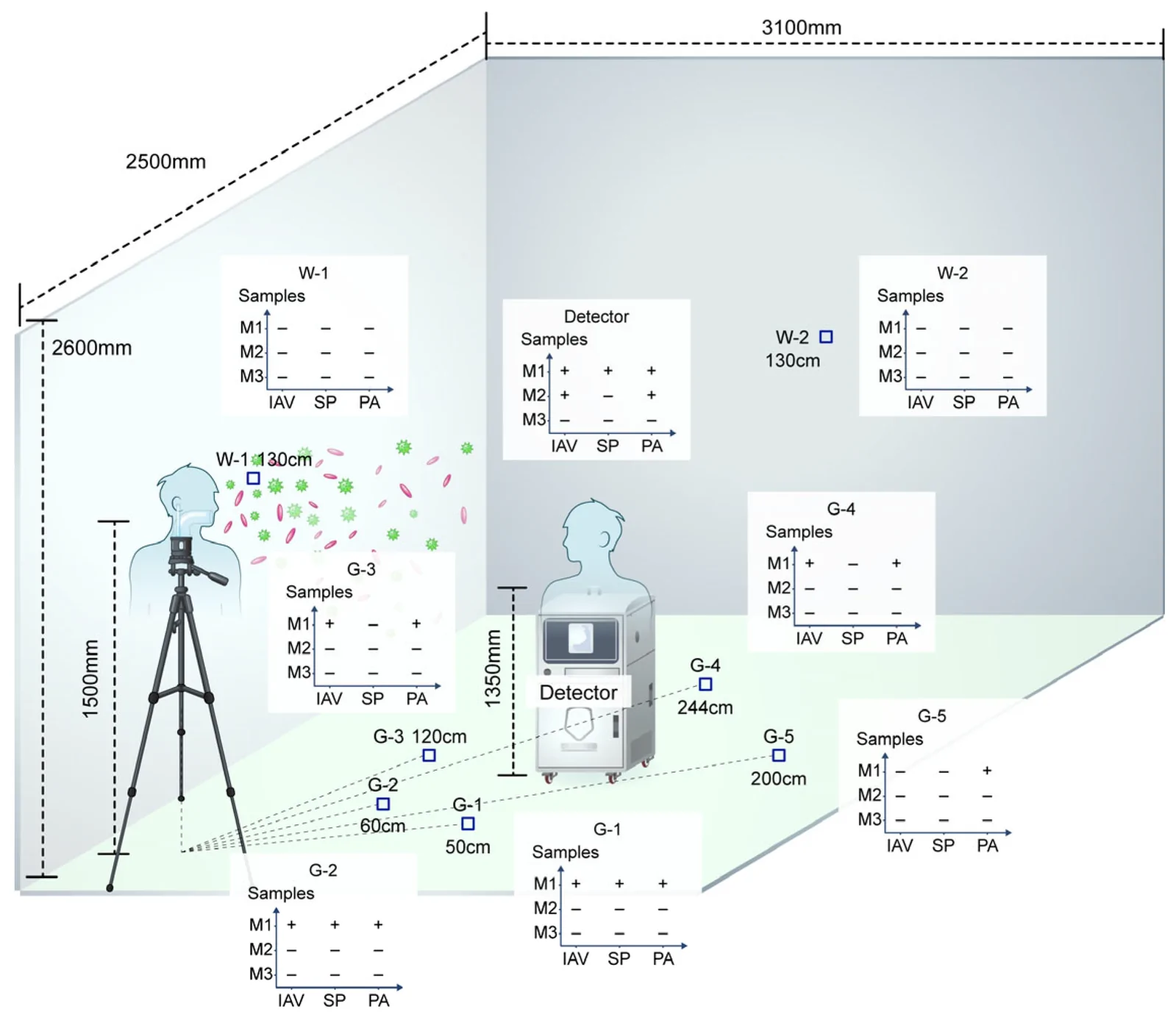
Layout of the aerosol chamber and comparison of APDS in situ air detection with environmental surface sampling at 20% relative humidity. The schematic illustrates the locations of the aerosol generator, the APDS detector, and the floor (G-1–G-5) and wall surface-sampling sites, with the corresponding sampling distances and heights indicated. Surface-sampling detection results for influenza A virus (IAV), *Streptococcus pneumoniae* (SP), and *Pseudomonas aeruginosa* (PA) are shown under three aerosol concentration conditions (M1–M3). All experiments shown in this figure were conducted at 20% relative humidity (RH). “+” and “−” indicate positive and negative detection results, respectively. APDS, autonomous pathogen detection system.

**Figure 3:**
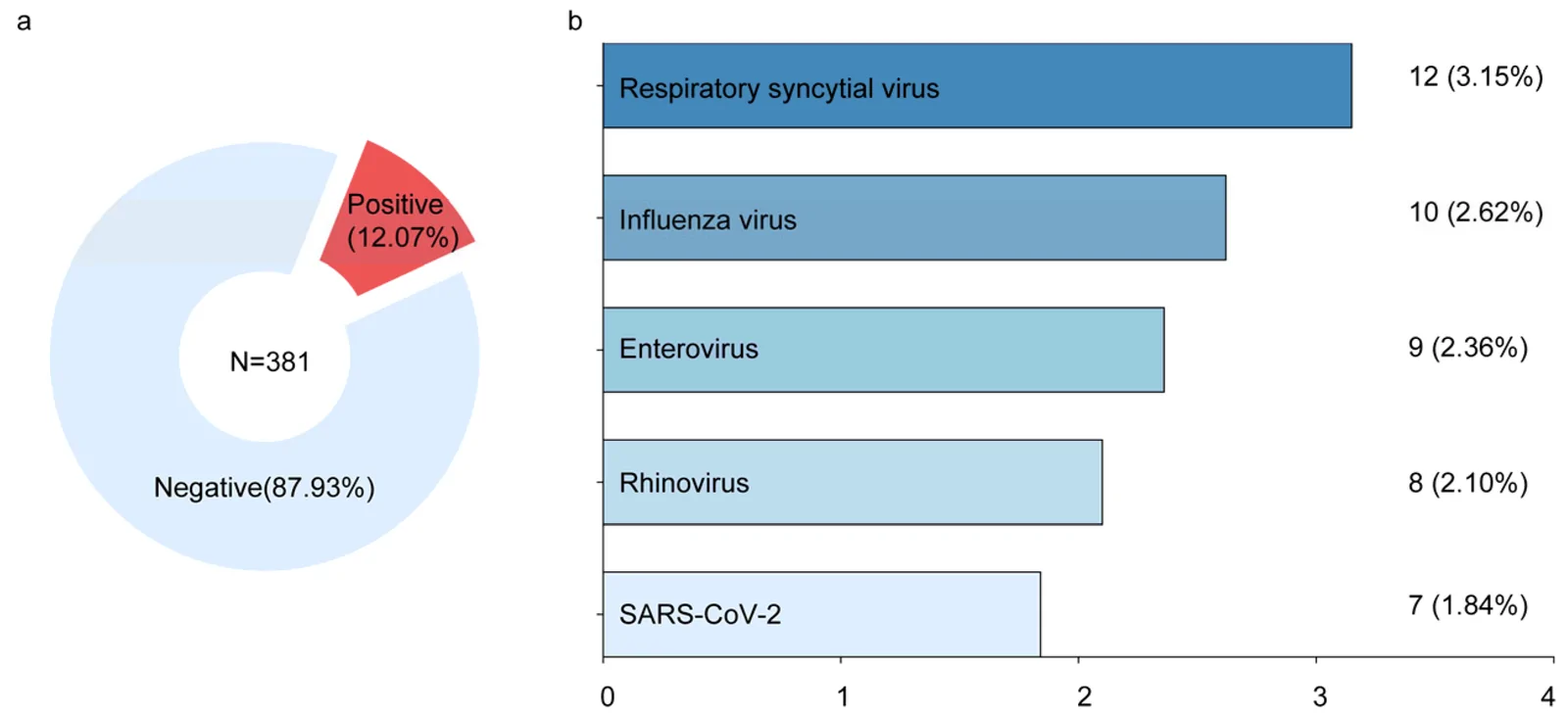
Summary of respiratory pathogen detections during 52 weeks of APDS field monitoring. (**a**) Overall distribution of positive and negative results among 381 autonomously triggered tests. (**b**) Numbers and overall positivity rates of detections for respiratory syncytial virus, influenza virus, enterovirus, rhinovirus, and SARS-CoV-2. The positivity rate for each pathogen was calculated using the total number of APDS-triggered tests (*n* = 381) as the denominator. No triggered test contained more than one pathogen-positive result; therefore, the 46 pathogen-specific positive results corresponded to 46 distinct positive triggered tests. APDS, autonomous pathogen detection system.

**Figure 4:**
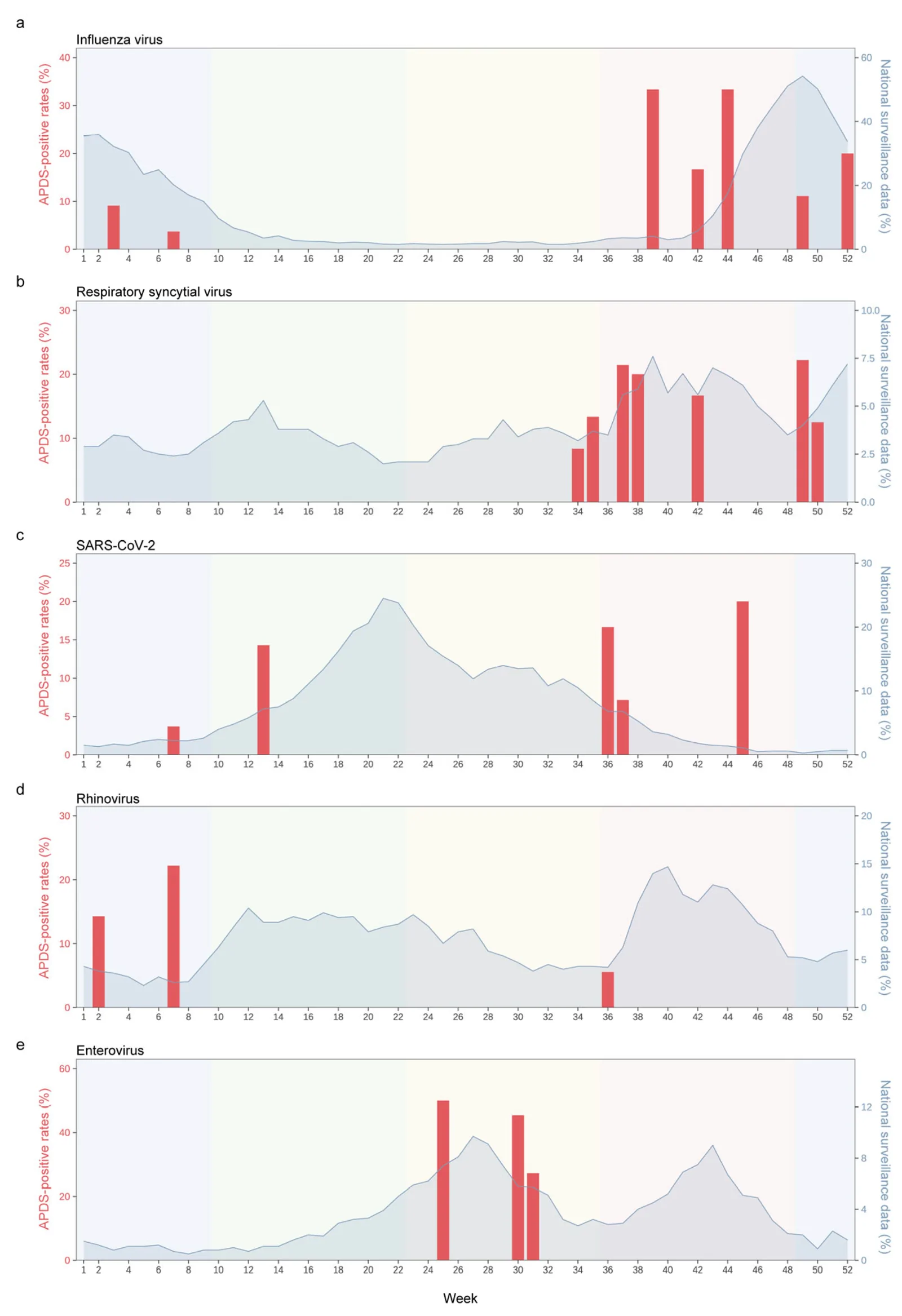
Weekly temporal patterns of APDS environmental surveillance and national sentinel surveillance for five respiratory pathogens during weeks 1–52 of 2025. Panels show (**a**) influenza virus, (**b**) respiratory syncytial virus, (**c**) SARS-CoV-2, (**d**) rhinovirus, and (**e**) enterovirus. Red bars represent weekly APDS positivity rates (left *y*-axis), whereas the blue line and shaded area represent weekly positivity rates from national sentinel surveillance of acute respiratory infectious diseases (right *y*-axis). The colored background bands indicate seasonal periods: blue, winter; light green, spring; light yellow, summer; and light red, autumn. APDS, autonomous pathogen detection system.

**Figure 5:**
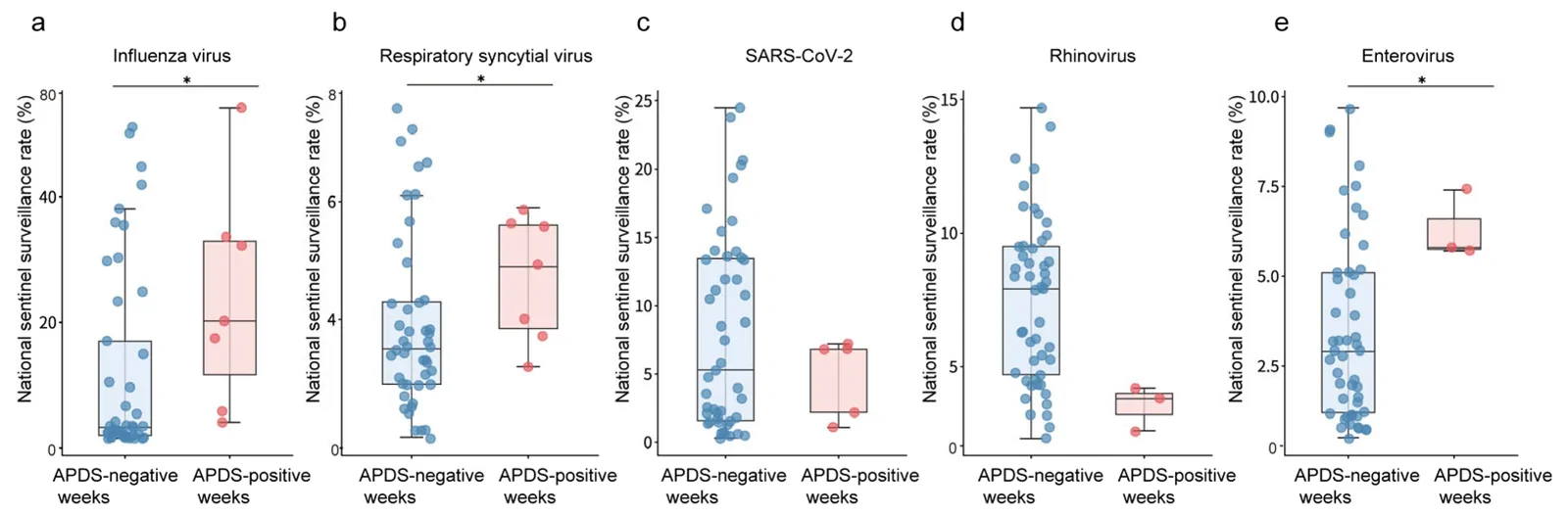
Comparison of national sentinel surveillance positivity rates between APDS-negative and APDS-positive weeks for five respiratory pathogens. Monitoring weeks were classified as APDS-negative or APDS-positive according to whether the corresponding pathogen was detected by APDS: (**a**) influenza virus, (**b**) respiratory syncytial virus, (**c**) SARS-CoV-2, (**d**) rhinovirus, and (**e**) enterovirus. Each dot represents an individual monitoring week. Blue and red symbols indicate APDS-negative and APDS-positive weeks, respectively. Boxes represent the interquartile range, horizontal lines within the boxes indicate the median, and whiskers show the distribution of values beyond the interquartile range. Differences between APDS-negative and APDS-positive weeks were assessed using a two-sided Mann–Whitney U test. * *p* < 0.05. APDS, autonomous pathogen detection system.

**Table 1:** Concentrations of quality-control materials at different dilution levels.

Dilution Level	M Group	Influenza A Virus (TCID_50_/mL)	*Streptococcus pneumoniae* (copies/mL)	*Pseudomonas aeruginosa* (copies/mL)	CL Group	SARS-CoV-2 (TCID_50_/mL)	*Legionella pneumophila* (copies/mL)
1	M1	2.5 × 10^5^	5.30 × 10^6^	1.20 × 10^5^	CL1	5.13 × 10^5^	5.10 × 10^6^
2	M2	2.5 × 10^4^	5.30 × 10^5^	1.20 × 10^4^	CL2	5.13 × 10^4^	5.10 × 10^5^
3	M3	2.5 × 10^3^	5.30 × 10^4^	1.20 × 10^3^	CL3	5.13 × 10^3^	5.10 × 10^4^

Note: M1–M3 and CL1–CL3 represent three serial dilution levels of the corresponding quality-control materials.

**Table 2:** Detected concentrations of aerosolized respiratory pathogens at different relative humidity levels.

Concentrations	Target	Theoretical Aerosol Concentration in Chamber	Detected Concentration at 20% RH	Detected Concentration at 70% RH ^1^
M2	Influenza A virus	3.75 × 10^4^ TCID_50_/m^3^	25.12 ± 1.63 TCID_50_/mL	21.31 ± 0.46 TCID_50_/mL
M1	*Streptococcus pneumoniae*	7.95 × 10^5^ copies/m^3^	7408.83 ± 920.13 copies/mL	5368.47 ± 402.13 copies/mL
M2	*Pseudomonas aeruginosa*	1.80 × 10^4^ copies/m^3^	42.24 ± 2.61 copies/mL	25.81 ± 5.69 copies/mL

^1^ Indicates a significant difference compared with 20% RH (*p* < 0.05).

**Table 3:** Theoretical aerosol concentrations and detected concentrations of SARS-CoV-2 and *Legionella pneumophila* at 20% relative humidity.

Concentrations	Target	Theoretical Aerosol Concentration in Chamber	Detected Concentration at 20% RH
CL2	SARS-CoV-2	7.70 × 10^3^ TCID_50_/m^3^	11.18 ± 1.88 TCID_50_/mL
CL2	*Legionella pneumophila*	7.65 × 10^4^ copies/m^3^	856.31 ± 18.22 copies/mL

**Table 4:** Spearman correlations between APDS measurements and surveillance data at different time lags.

Pathogen	Lag −3	Lag −2	Lag −1	Lag 0	Lag +1	Lag +2	Lag +3
Influenza virus	0.265	0.307 *	0.314 *	0.321 *	0.275	0.313 *	0.371 *
RSV	0.237	0.154	0.135	0.245	0.378 *	0.471 **	0.487 **
SARS-CoV-2	−0.081	−0.086	−0.081	−0.090	−0.132	−0.134	−0.137
Rhinovirus	−0.267	−0.270	−0.282	−0.331	−0.262	−0.098	−0.034
Enterovirus	0.327	0.332	0.281	0.265	0.260	0.215	0.121

Values represent Spearman’s ρ. *p* values for the seven lag comparisons within each pathogen were adjusted using the Benjamini–Hochberg false discovery rate method. * q < 0.05; ** q < 0.01.

**Table 5:** Comparison of the technical characteristics of two respiratory pathogen surveillance systems.

Feature	APDS Environmental Aerosol Surveillance	National Sentinel Surveillance of Acute Respiratory Infectious Diseases
Monitoring target	Bioaerosols and nucleic acid signals of target respiratory pathogens in ambient air	Patients with influenza-like illness presenting to outpatient services
Sampling source	Aerosols in a hospital outpatient hall	Clinical respiratory specimens, such as nasopharyngeal and oropharyngeal swabs, from patients seeking medical care
Denominator	Number of triggered tests during the same period	Total number of respiratory specimens from influenza-like illness cases tested at sentinel hospitals during the same period
Data reporting	Automatically uploaded to the cloud platform after testing and available for remote review	Entered and reported to the surveillance system of the Chinese Center for Disease Control and Prevention after laboratory testing

## Data Availability

The original contributions presented in this study are included in the article/Supplementary Materials. Further inquiries can be directed to the corresponding author.

## Supplementary Materials

The following supporting information can be downloaded at: https://www.mdpi.com/article/10.3390/microorganisms14102192/s1, Table S1. Summary of aerosol chamber experimental conditions. Table S2. Environmental monitoring sites in the aerosol chamber. Table S3. Detection results of five targets at different theoretical concentrations. Table S4. Weekly numbers of APDS-triggered tests and pathogen-specific positive detections during the 52-week field monitoring period.
