# Supplementary material for: An Autonomous Pathogen Detection System for Assessing Temporal Associations with Respiratory Pathogen Activity
Source: Microorganisms. 2026 Oct 1;14(10):2192. doi: 10.3390/microorganisms14102192 (PMC13707842; doi:10.3390/microorganisms14102192)
Supplement: Supplementary file 1 [file microorganisms-14-02192-s001.zip › microorganisms-4553231-supplementary.pdf]

Determination of the Minimum Consistently Detectable Concentration of Respiratory Pathogen Aerosols

An APDS unit was placed at the center of the aerosol chamber for testing. The experimental conditions are summarized in Table S1. Temperature was kept constant, while relative humidity was set at 20% or 70%; the nebulization flow rate, nebulization time, and aerosol mixing time were kept identical across conditions. The microbial suspensions were serially diluted 10-fold in PBS, and 9 mL of each dilution was added to the aerosol generator. The generator was connected to the laboratory compressed-air supply for nebulization. After aerosol mixing, APDS automatically performed air sampling and in situ detection. Each concentration condition was tested in triplicate.

The theoretical aerosol concentration in the chamber was calculated as follows: $C = (c \times v)/V$,

where C is the theoretical aerosol concentration in the chamber, c is the concentration of the target pathogen in the suspension, v is the volume of suspension nebulized by the aerosol generator, and V is the chamber volume (20 m^3). Particle sedimentation was not included in this calculation.

Table S1. Summary of aerosol chamber experimental conditions

Group	Generator contents	Temp. (°C)	RH (%)	Nebulization flow (L/min)	Nebulization time (s)	Mixing time (s)	Sampling volume (L)	Replicates
Group 1	M1	25 °C	20%	30	300	600	2000	3
Group 2	M1	25 °C	70%	30	300	600	2000	3
Group 3	M2	25 °C	20%	30	300	600	2000	3
Group 4	M2	25 °C	70%	30	300	600	2000	3
Group 5	M3	25 °C	20%	30	300	600	2000	3
Group 6	M3	25 °C	70%	30	300	600	2000	3

After each run, swab samples were collected from environmental monitoring sites in the chamber. As summarized in Table S2, five floor sites were located at different distances from the aerosol generator, and two wall sites were designated; the sampling area at each site was 25 cm^2. Surface detection results were compared with the corresponding air detection results. After each experiment, the chamber was disinfected by ultraviolet irradiation for 2 h and ventilated for 4 h. The floor and walls were then disinfected with a 0.2% chlorine-containing disinfectant for 30 min and wiped with clean water. After

disinfection, floor and wall surfaces were resampled and tested to verify decontamination before the next experiment was performed.

Table S2. Environmental monitoring sites in the aerosol chamber

Site ID	Surface	Position
G-1	Floor site 1	50 cm from the point directly beneath the aerosol generator
G-2	Floor site 2	60 cm from the point directly beneath the aerosol generator
G-3	Floor site 3	120 cm from the point directly beneath the aerosol generator
G-4	Floor site 4	244 cm from the point directly beneath the aerosol generator
G-5	Floor site 5	200 cm from the point directly beneath the aerosol generator
W-1	Wall site 1	130 cm above the floor
W-2	Wall site 2	130 cm above the floor

Standard Curve Construction

Standard curves were constructed using ten-fold serial dilutions, with each concentration tested in triplicate by quantitative real-time PCR. The standard curve equations were $y = -3.48x + 39.05$ ($R^2 = 0.999$, $E = 93.98$) for influenza A virus, $y = -3.42x + 47.46$ ($R^2 = 0.999$, $E = 96.01$) for *Streptococcus pneumoniae*, $y = -3.53x + 37.89$ ($R^2 = 1.000$, $E = 92.08$) for *Pseudomonas aeruginosa*, $y = -3.29x + 37.67$ ($R^2 = 0.996$, $E = 101.20$) for SARS-CoV-2, and $y = -3.46x + 44.56$ ($R^2 = 1.000$, $E = 94.59$) for *Legionella pneumophila*. All standard curves showed good linearity over the tested concentration ranges.

Table S3. Detection results of five targets at different theoretical concentrations

Target	Theoretical concentration 1 in chamber	Ct value	Theoretical concentration 2 in chamber	Ct value	Theoretical concentration 3 in chamber	Ct value
Influenza A virus $TCID_{50}/m^3$	3.75×10^5	30.82/ 31.01/ 30.95	3.75×10^4	34.25/ 34.07/ 34.22	3.75×10^3	-
Streptococcus pneumoniae copies/m^3	7.95×10^5	34.44/ 34.72/ 34.81	7.95×10^4	-	7.95×10^3	-
Pseudomonas aeruginosa copies/m^3	1.80×10^5	32.06/ 32.25/ 32.56	1.80×10^4	34.77/ 34.15/ 34.65	1.80×10^3	-

SARS-CoV-2 $TCID_{50}/m^3$	7.70×10^4	31.74/ 31.28/ 31.34	7.70×10^3	34.41/ 33.97/ 34.32	7.70×10^2	-
Legionella pneumophila copies/m^3	7.65×10^5	31.68/ 31.55/ 31.37	7.65×10^4	34.45/ 34.39/ 34.40	7.65×10^3	-

Weekly numbers of APDS-triggered tests

To provide the denominators underlying the weekly APDS positivity rates, the total number of APDS-triggered tests and the number of pathogen-specific positive detections for each week are summarized in Table S4. Weekly positivity rates for each pathogen were calculated as the number of positive detections divided by the total number of APDS-triggered tests performed during the corresponding week.

Table S4. Weekly numbers of APDS-triggered tests and pathogen-specific positive detections during the 52-week field monitoring period.

Week	Influenza positive	SARS-CoV-2 positive	RSV positive	Rhinovirus positive	Enterovirus positive	No. of triggered tests	Failure reason
1	0	0	0	0	0	2	
2	0	0	0	1	0	7	
3	2	0	0	0	0	22	
4	0	0	0	0	0	14	
5	0	0	0	0	0	0	Power failure
6	0	0	0	0	0	0	Power failure
7	1	1	0	6	0	27	
8	0	0	0	0	0	13	
9	0	0	0	0	0	0	Device Maintenance
10	0	0	0	0	0	2	
11	0	0	0	0	0	4	
12	0	0	0	0	0	2	
13	0	1	0	0	0	7	
14	0	0	0	0	0	7	
15	0	0	0	0	0	12	
16	0	0	0	0	0	8	
17	0	0	0	0	0	9	
18	0	0	0	0	0	3	
19	0	0	0	0	0	5	
20	0	0	0	0	0	1	
21	0	0	0	0	0	2	
22	0	0	0	0	0	5	
23	0	0	0	0	0	10	
24	0	0	0	0	0	9	

25	0	0	0	0	1	2	
26	0	0	0	0	0	4	
27	0	0	0	0	0	6	
28	0	0	0	0	0	9	
29	0	0	0	0	0	12	
30	0	0	0	0	5	11	
31	0	0	0	0	3	11	
32	0	0	0	0	0	4	
33	0	0	0	0	0	14	
34	0	0	1	0	0	12	
35	0	0	2	0	0	15	
36	0	3	0	1	0	18	
37	0	1	3	0	0	14	
38	0	0	2	0	0	10	
39	2	0	0	0	0	6	
40	0	0	0	0	0	0	Power failure
41	0	0	0	0	0	1	
42	1	0	1	0	0	6	
43	0	0	0	0	0	2	
44	2	0	0	0	0	6	
45	0	1	0	0	0	5	
46	0	0	0	0	0	5	
47	0	0	0	0	0	0	Device Maintenance
48	0	0	0	0	0	10	
49	1	0	2	0	0	9	
50	0	0	1	0	0	8	
51	0	0	0	0	0	5	
52	1	0	0	0	0	5	
